# Discrimination against international medical graduates in the United States residency program selection process

**DOI:** 10.1186/1472-6920-10-5

**Published:** 2010-01-25

**Authors:** Norman A Desbiens, Humberto J Vidaillet

**Affiliations:** 1 12301 Creek Hollow Lane, Soddy Daisy, Tennessee, USA; 2 Marshfield Clinic Research Foundation, 1000 N. Oak Avenue, Marshfield Wisconsin, USA

## Abstract

**Background:**

Available evidence suggests that international medical graduates have improved the availability of U.S. health care while maintaining academic standards. We wondered whether studies had been conducted to address how international graduates were treated in the post-graduate selection process compared to U.S. graduates.

**Methods:**

We conducted a Medline search for research on the selection process.

**Results:**

Two studies provide strong evidence that psychiatry and family practice programs respond to identical requests for applications at least 80% more often for U.S. medical graduates than for international graduates. In a third study, a survey of surgical program directors, over 70% perceived that there was discrimination against international graduates in the selection process.

**Conclusions:**

There is sufficient evidence to support action against discrimination in the selection process. Medical organizations should publish explicit proscriptions of discrimination against international medical graduates (as the American Psychiatric Association has done) and promote them in diversity statements. They should develop uniform and transparent policies for program directors to use to select applicants that minimize the possibility of non-academic discrimination, and the accreditation organization should monitor whether it is occurring. Whether there should be protectionism for U.S. graduates or whether post-graduate medical education should be an unfettered meritocracy needs to be openly discussed by medicine and society.

## Background

The United States owes a huge debt of gratitude to its physicians who graduated from non-U.S. medical schools [1]. Despite this, medical educators have sometimes seemed to be embarrassed by the presence of these residents in their programs. For example, in a recent article that praises international medical graduates (IMGs) one leader states that his program chooses that IMGs comprise 10 percent of his residents - a quota system that belies his later assertion that "U.S. academic medicine is ... a classic meritocracy" [2].

The proportion of IMGs practicing in the U.S. is considerable. About one-quarter of practicing U.S. physicians are IMGs, up from 15 percent in 1967 and 6.3 percent in 1959. In 2004, 28 percent of the residency cohort was represented by IMGs, and more so in some specialties, such as psychiatry and nephrology [3]. The proportion of U.S. physicians who themselves are immigrants or who are the children or grandchildren of immigrants is even greater [4]. This should not be surprising. The U.S. is a nation of immigrants that has always been dependent on those from other countries to make it an economic and intellectual powerhouse.

Recent studies indicate that medical educators, perhaps because of improved information from the U.S. Medical Licensing Exam (USMLE) and the Educational Commission for Foreign Medical Graduates (ECFMG), are becoming better at selecting and educating IMGs. For example, since 1995 non-U.S. graduates have outperformed U.S. graduates (USMGs) on the In-Training Examination [5].

Physicians from other countries have enriched U.S. medicine clinically, scientifically and culturally [6]. Relying on other countries to partially educate about a quarter of its physicians has saved the U.S. a huge amount of money. In 2002, instructional costs ranged from about $48,000 to $51,000 and educational resources $80,000 to $105,000 per student per year [7]. Medical educational organizations have recently argued that the U.S. should increase the number of U.S. students, and many allopathic and osteopathic schools are in the process of so doing. The major rationale that they have used has been to meet the increased demand for medical care that a burgeoning population will bring, not to replace non-U. S. graduates in residency programs [8].

Despite the benefits that IMGs have brought to the U.S., we have perceived discrimination against IMGs during the residency selection process. We wondered whether there was research evidence in the medical literature to support our impression.

## Methods

On 5-27-09 we queried Medline using the following search string using MESH headings: "biomedical research" AND "Education, Medical, Graduate" AND "Foreign Medical Graduates". This search produced one irrelevant article. We then used the following string: (international OR foreign) AND ((graduate OR postgraduate) AND "medical education"). We found 1543 papers from 1961 to the present. We manually reviewed this listing for scientific studies of the recruitment process. We found two papers that used paired data designs to determine whether U.S. graduate medical educational programs responded to requests for applications by U.S. and international medical graduates differently. We then used the "related articles" feature on each of these papers which led us to another paper that surveyed surgical program directors (a cross-sectional study design) about international medical graduates which we used in the discussion section. We searched the reference lists of these papers and found no additional research articles.

We report the author's statistics for the two studies that requested applications. They used McNemar's chi-square for correlated proportions or the continuity-corrected McNemar's chi-square. In addition to the authors' calculations, we calculated relative response rates. For dichotomous variables from the survey study, we calculated 95% binomial confidence intervals using the binconf function in Harrell's Hmisc library in S-Plus (Insightful Corporation; Seattle, WA).

## Results

We found three relevant studies. Two were directed at the application process and used similar paired data designs, while a third surveyed surgical residency program directors for their perceptions about IMGs (cross-sectional study design).

One study sent applications to 146 family practice residency programs randomly selected from the 384 programs in the *Directory of Graduate Medical Education Programs, 1991-1992 *(38% sampled) [9]. The letters requested information and an application. All letters were identical except that the author of the first set was described as "a foreign medical graduate" while the author of the second was described as "a fourth-year medical student at the University of Nebraska Medical Center". Pseudonyms were used and surnames were selected that "would not suggest any particular ethnic group". Only a first initial was used to eliminate the possibility of gender bias. The letters from the IMG were sent first, and those from the USMG followed one week later. Of the 146 requests, 143 were received by programs.

When analyzed at 6 weeks by any response, 102 programs (71%) responded to the fourth-year medical student and 57 (40%) to the foreign graduate (relative response, U.S. medical student to foreign graduate: 1.8). Of the 46 programs responding to both, 9 required the foreign graduate to meet standards that exceeded requirements set by the ECFMG. When analyzed by reception of application forms, 39 programs sent applications to both (27%), 60 to only the U.S. medical student applicant (42%), 10 to only the foreign graduate (7%) and 34 to neither (24%) (relative application response, U.S. medical student to foreign graduate: 2.0; p < .01).

The second study sent identical requests (details not provided) for a program application to 193 psychiatry residency training programs, omitting those in Michigan since the persons requesting applications were enrolled in a Michigan program. The letters differed in only two respects: the names of the writers (one "American" and one "Pakistani") and the medical schools from which they graduated (Wayne State University School of Medicine and King Edward Medical College). Letters were sent one week apart. Five programs reported they were closed, leaving 188 for analysis.

When analyzed by any response, 99 programs (53%) responded to both applicants, 60 only to the USMG (32%), 6 only to the IMG (3%) and 23 to neither requestor (12%) (p < .001; relative response, USMG to IMG: 1.5). When analyzed by reception of application forms, the USMG received 159 responses with application forms (85% response rate) while the IMG received 87 responses with application forms (46%) (p < .001; relative application response, USMG to IMG: 1.8) [10]. The authors also report that in the year prior to their study, psychiatry residency slots remained empty, with only 84 percent of available positions filled.

The third study surveyed all 283 members of the Association of Program Directors of Surgery in 2007 [11]. Of these, they determined that 261 were active at the time of the survey and this was their targeted study population; 125 directors responded (48%) and 112 were analyzed. Most of the program directors were male (95%). They were 52 years of age (range: 37, 71 years) on average and their median tenor as program directors was 7 years; 90% reported being USMGs and 8% IMGs; and 49% were university-based and 47% community-based.

In response to five-point Likert-scale questions, 69 (59, 77) (95% binomial confidence interval) percent of directors strongly agreed, agreed or were neutral to the statement that on standardized exams IMGs perform as well as USMGs and 79 (70, 85) percent strongly agreed, agreed or were neutral to the statement that surgical skill level, as measured by performance in the operating room, is equal or better for IMGs compared to USMGs. For the statement, "In reality, all things being equal, our program would rather offer positions to USMGs than to IMGs", 97 (92, 99) percent agreed or were neutral (strongly agreed [47%], agreed [40%], neutral [10%]). In response to a yes or no question, 18 (11, 26) percent of directors answered that they had felt external pressure not to rank a better qualified IMG over a USMG and 71 percent felt that IMGs are discriminated against.

## Discussion

Despite the difficulty of performing research on discrimination, we were able to find two studies conducted with family practice and psychiatry programs that reported similar methods and findings. In addition, a survey of directors of surgical residency program reported that more than 70 percent of directors believed IMGs were discriminated against in the selection process and nearly 20 percent reported that they had been pressured to discriminate against IMGs in favor of USMGs.

The paired-study technique used by two of the studies is a strong design and has a long biometrical application history. A large random sample of programs was used in one study but sampling bias was not an issue in the other, since it used an enumerative design: all programs in the specialty, with a few feasibility exceptions, were studied. The effect sizes in both studies were large and consistent. The differences in responses and responses to requests for applications in each study were large and statistically significantly biased in favor of USMGs over IMGs by a 50 to 100 percent margin.

Nonetheless, it would have been helpful to see similar studies in other larger specialty residency programs such as internal medicine and surgery, though inferences to the latter specialty programs are strengthened by the findings from the surgical program directors' survey. A listing of the details of the request letters or a sample figure would have been helpful. A higher response to the survey would have strengthened point estimates on the questions, but the findings are disconcerting despite this limitation. For example, if all of the non-responders had answered that they did not feel that there was discrimination against IMGs, 31 (25, 37) percent of directors would have agreed with this statement -- still a worrisome figure. Finally, a more extensive search using sources in addition to Medline might have discovered other relevant studies.

Our findings provide scientific evidence to bolster opinions in the medical literature that there is discrimination against IMGs in the selection process. This bias could be operationalized in three ways: categorical refusal to consider non-U.S. applicants, quota systems and hierarchical two-rank systems. In a quota system, a program determines the percentage of IMGs that it will allow. Quotas are facilitated by the present system that allows IMGs (and osteopathic and former USMGs) to be taken into a program outside the National Resident Matching Program (NRMP). A program may choose a certain number of IMGs before the match to meet its quota and then rank only USMGs for the remaining approved spots. A hierarchical two-rank system works by ranking USMGs first, then IMGs, regardless of qualifications.

Part of the bias against IMGs by residency programs in the past may have been evaluative bias. It had been very difficult to ascertain whether IMGs were adequately trained or prepared for U.S. residency programs [12]. However, changes made by the ECFMG have largely eliminated this problem. IMGs now take the same medical knowledge examination as USMGs (the USMLE), must all pass a standardized language exam (TOEFL or the former ECFMG English test), and must travel to the U.S. to prove their history and physical exam abilities (ECFMG CSA [clinical skills assessment]). Standardized examinations such as the USMLE are being increasingly recognized as valid indicators of professional success [13]. Most programs also require selected applicants to come for an interview to vet their communication and interpersonal skills. In fact, in many ways it is now easier to evaluate IMGs than many graduates of U.S. osteopathic schools who often do not take the USMLE.

If evaluative bias is no longer a factor, then why would U.S. programs discriminate against IMGs during the application process? Possibilities include additional educational burden and costs imposed on programs by IMGs, conflict over goals for medical and post-graduate medical education, protectionism for USMGs, concerns about image, administrative simplification, and discrimination based on xenophobia, country of origin, chauvinism, or other factors.

There is no doubt that IMGs impose an extra educational burden on U.S. residency programs, especially in the first year of residency. These graduates have to be acculturated to the U.S. and its health care and laws, the epidemiology of disease in the U.S., patient-physician cooperative decision making, the willingness and ability of the U.S. to spend much more to improve quality and length of life than their home countries, and perhaps evidence-based medicine, AIDS care in the U.S. and cultural competence, among others. These knowledge deficits are not trivial and take real resources in the form of special curriculum and faculty time to resolve. However, experience indicates that they can be expunged [14].

We suspect that post-graduate medical education has been protectionistic because educators conflate academic considerations with social policy. If U.S. residency programs were pure meritocracies, there should be no categorical refusal to consider IMGs and no quotas, and USMGs and IMGs should be intercalated in match lists of all programs. Applicants from all countries, including the U.S., would be ranked solely on individual merit. This approach would allow the best and brightest from throughout the world to compete for U.S. residency positions.

Those who favor protectionism might argue that federal or state legislatures made a social contract with their citizens that if they undertook the rigors, expense and, oftentimes, indebtedness of medical education, they would be guaranteed a residency position in this country. The available evidence suggests that this is the implicit policy followed by the majority of U.S. programs. Whether such an approach leads to the best physicians for a country needs to be studied. U.S. medical organizations need to lead the discussion on these issues and develop explicit policies for selecting IMGs.

Medical schools' perceptions of the *raison d'être *of their post-graduate programs may conflict with those of residency program leadership. If a primary purpose of the residency programs is perceived to be assisting medical school graduates to obtain U.S. residency positions, residency programs might use different selection strategies than if their goal were academic excellence. For example, they might rank U.S. students in the tenth percentile on the USMLE ahead of IMGs in the ninety-ninth percentile or not rank the latter at all. This potential conflict of interest between residency program and sponsoring medical schools needs to be openly discussed.

Medical educators may want to limit the number of IMGs in their programs because of general perceptions among faculty, residents, U.S. applicants and even non-U.S. applicants that programs with IMGs are inferior [15]. They may be concerned about image or that image will affect recruiting of U.S. applicants. To combat these perceptions, U.S. medicine should promote objective standards for applicants to use in evaluating the quality of residency programs that do not consider the medical school or country of origin of residents. These criteria could include board passage rate, faculty research, resident presentations and research, aggregate USMLE scores, resident success in obtaining fellowships or positions, and resident satisfaction surveys, *inter alia*. Once these sources of bias have been eliminated, there remains the likelihood that discrimination based on xenophobia, racism, chauvinism or other factors is operative.

At this point in a manuscript it is customary for authors to suggest that further studies be undertaken to strengthen and extend existing findings. We doubt that this would be possible for at least the studies that used paired comparison. In the nearly fifty years of articles analyzed by our search, the three studies that we identified were conducted in an eight year period between 1994 and 2002. We could not find any indication that the studies were approved by an institutional review board and they used some degree of deception in order to gather data. Since 2002, very little has changed to encourage residency programs to use just and unbiased methods to deal with all applicants.

Much can be done to prevent or eliminate discrimination against IMGs. Interested stakeholders could convene task forces to deal with the overarching issue of meritocracy and social policy and medical schools' posture towards post-graduate education, and give program directors clear guidelines for selecting applicants. Such transparent policies could assist program directors in dealing with the deluge of IMG applicants, so that categorical refusal to considers these applicants is not used for administrative simplification. Research studies could be undertaken with the assistance of the NRMP to better understand and monitor the occurrence of categorical non-ranking of non-U.S. applicants and two-tiered hierarchal match lists. U.S. medical schools and specialty organizations could explicitly mention IMGs in their diversity statements and actively monitor perceptions of discrimination with confidential questionnaires. (To our knowledge, the American Psychiatric Association is the only medical group that has published an explicit statement opposing discrimination against IMGs, though it does not specifically refer to residency selection [16].) The Liaison Committee on Medical Education could require and monitor these activities. The Accreditation Council for Graduate Medical Education could add similar statements to its common requirements and monitor perceptions in its surveys to residents, and develop a standardized list of objective criteria for applicants to use to evaluate residency programs. Requiring IMGs to enter the NRMP would effectively eliminate quota systems.

## Conclusions

It is time for the U.S. to re-examine its residency selection process and develop explicit and just selection policies for all applicants. Farra has expressed it aptly: "We have a duty to help this group that is so important. When you get a chance, ask your IMG colleagues about their stories and struggles to become accepted in this country. Let us welcome more of them into our large House of Medicine so that we can continue to make it better for everyone, especially our patients" [17]. A recent review concludes that, "the reduction of prejudice is not just a social nicety. Prejudice and discrimination are profoundly harmful to individuals and society as a whole." [18]. More openness in and monitoring of the residency selection process would help propagate the message that U.S. medicine abjures discrimination. The available evidence suggests otherwise and supports the contention of a prominent U.S. medical educator that, "there is little doubt that a bias against IMGs has existed within graduate medical training in the U.S." [19].

## Competing interests

The authors declare that they have no competing interests.

## Authors' contributions

ND did the literature search and wrote the manuscript drafts. HV was involved in many discussions and critical revisions. Both authors read and approved the final manuscript.

## Authors' information

ND recently retired from the Chattanooga Unit of the University of Tennessee College of Medicine where he was Chair and Professor of Medicine. HV is Clinical Professor of Medicine at the University of Wisconsin Medical School and Director of the Marshfield Clinic Research Foundation. They represent a combined 60 years of experience as medical educator and have served either as members of residency program selection committees, program director of residency programs or a chair of a department of medicine.

## Pre-publication history

The pre-publication history for this paper can be accessed here:

http://www.biomedcentral.com/1472-6920/10/5/prepub

## References

[B1] Gastel B Impact of International Medical Graduates on U.S. and Global Health Care: summary of the ECFMG 50th anniversary invitational conference Acad Med 2006 81 S3 S6 10.1097/01.ACM.0000243340.89496.43 17086042

[B2] Centor R What I Have Learned from IMGs SGIM Forum 2007 30 3 12

[B3] Akl EA Mustafa R Bdair F Schunemann HJ The United States Physician Workforce and International Medical Graduates: Trends and Characteristics J Gen Intern Med 2007 22 264 268 10.1007/s11606-006-0022-2 17356997 PMC1824721

[B4] Brown D At Med Schools, A New Degree of Diversity: Classes Reflect a Foreign Flavor Washington Post 2007

[B5] Garibaldi RA Subhiyah R Moore ME Waxman H In-Training Examination in Internal Medicine: an analysis of resident performance over time Ann Intern Med 2002 137 505 10 12230352 10.7326/0003-4819-137-6-200209170-00011

[B6] Stephan PE Levin SG Exceptional contributions to US science by the foreign-born and foreign-educated Pop Research and Pol Rev 2001 20 59 79 10.1023/A:1010682017950

[B7] Report 2 of the Council on Medical Education (I-00): Medical school financing, tuition and student debt http://www.ama-assn.org/ama1/pub/upload/mm/15/cme_report_2_i00.doc

[B8] Salsberg E Grover A Physician workforce shortages: implications and issues for academic health centers and policymakers Acad Med 2006 81 782 787 10.1097/00001888-200609000-00003 16936479

[B9] Nasir LS Evidence of discrimination against international medical graduates applying to family practice residency programs Fam Med 1994 26 625 9 7859953

[B10] Balon R Mufti R Williams M Riba M Possible discrimination in recruitment of psychiatry residents? Am J Psych 1997 154 1608 1609 10.1176/ajp.154.11.1608 9356575

[B11] Moore RA Rhodenbaugh EJ The unkindest cut of all: Are international medical school graduates subjected to discrimination by general surgery residency programs? Curr Surg 2002 59 228 236 10.1016/S0149-7944(01)00644-4 16093139

[B12] Tinsely JA McAlpine DE Another explanation for the apparent discrimination against international medical graduates by residency programs. (letter) Am J Psych 1999 156 496 497 10.1176/ajp.156.3.496a 10080577

[B13] Kuncel NR Hezlett SA Standardized tests predict graduate students' success Science 2007 315 1080 1081 10.1126/science.1136618 17322046

[B14] Horvath K Coluccio G Foy H Pellegrini C A program for successful integration of international medical graduates (IMGs) into U.S. surgical residency training Curr Surg 2004 61 492 498 10.1016/j.cursur.2004.06.011 15475104

[B15] Riley JD Hannis M Rice KG Are international medical graduates a factor in residency program selection? A survey of fourth-year medical students Acad Med 1996 71 381 386 10.1097/00001888-199604000-00017 8645405

[B16] The American Psychiatric Association Position Statement Discrimination Against International Medical Graduates Document Reference No. 200102 2001 Washington, D.C.: American Psychiatric Association

[B17] Farr PO The impact of international medical graduates in US health care Mich Med 2006 105 32 17069239

[B18] Christie DJ Dawes A Tolerance and solidarity J of Peace Psych 2001 7 131 142 10.1207/S15327949PAC0702_04

[B19] Waxman H Workforce reform, international medical graduates, and the in-training examination 1997 126 803 805 9148655 10.7326/0003-4819-126-10-199705150-00011

